# Ventricular Migration of Transcatheter Valve During Transcatheter Aortic Valve Replacement After Valve-Sparing Root Replacement: A Case Report

**DOI:** 10.1016/j.jscai.2026.105344

**Published:** 2026-04-30

**Authors:** Meenakshi Mandal, Mayra Guerrero, Conor Lane, Richard Daly, Juan A. Crestanello

**Affiliations:** a Department of Cardiovascular Surgery, Mayo Clinic, Rochester, Minnesota; b Department of Cardiovascular Medicine, Mayo Clinic, Rochester, Minnesota

**Keywords:** aortic root intervention, case report, complications, transcatheter aortic valve replacement, valve-sparing aortic root replacement

## Abstract

Transcatheter aortic valve replacement is increasingly used off-label for native aortic regurgitation in high surgical risk patients, although the absence of annular calcification—particularly after prior valve-sparing aortic root replacement—complicates valve anchoring and increases migration risk. We report a 66-year-old man with severe symptomatic aortic regurgitation after valve-sparing aortic root replacement and hemiarch repair who underwent transcatheter aortic valve replacement. Following deployment of a 29-mm Evolut FX+ valve (Medtronic), valve migration into the left ventricular outflow tract caused severe regurgitation and instability. A second 26-mm SAPIEN 3 Ultra valve (Edwards Lifesciences) was successfully implanted to stabilize the prosthesis, restoring competence and enabling uneventful recovery.

## Introduction

The rapid evolution of device technology and procedural techniques of transcatheter aortic valve replacement (TAVR) has expanded its indications beyond calcific aortic stenosis to include select patients with aortic regurgitation (AR)—particularly those at elevated surgical risk.^1^^,^^2^ However, TAVR in the setting of pure AR presents several unique technical and anatomical challenges,^3^ which are further amplified in patients with prior valve-sparing aortic root replacement (VSARR), where the aortic valve is reimplanted within a prosthetic Dacron graft. Although VSARR preserves native valve function and avoids long-term anticoagulation, it alters aortic root geometry and compliance, eliminating native landmarks that facilitate transcatheter heart valve (THV) fixation. In this setting, THV anchoring depends primarily on radial force, oversizing, and friction with the prosthetic graft. The absence of calcification and the noncompliant synthetic graft increase the risk of valve migration, which can cause acute severe regurgitation and rapid hemodynamic deterioration.^4^

Although surgical retrieval or repositioning may be necessary in some cases, second valve-in-valve implantation has emerged as an effective and less invasive bailout strategy.^5^^,^^6^

This approach can secure the initial device, restore valve competence, and avoid emergent surgical intervention—particularly valuable in patients with prior complex aortic root surgery and elevated operative risk.

In this report, we present a rare case of ventricular migration of a THV during TAVR for severe native AR in a patient with previous VSARR, successfully managed with implantation of a second valve.

This case underscores the procedural complexities of TAVR in noncalcified, surgically modified aortic roots and highlights the importance of bailout strategies for valve malposition.

## Case presentation

A 66-year-old man with a history of VSARR with ascending aorta and hemiarch replacement performed 4 years earlier for an ascending aortic aneurysm presented with New York Heart Association Class IV symptoms of worsening dyspnea on exertion for 6 months and decreased exercise tolerance.

Echocardiography revealed a trileaflet aortic valve with severe aortic regurgitation. There was an eccentric aortic valve regurgitant jet with possible prolapse of the right coronary cusp. He had preserved left ventricular ejection fraction of 67% and normal filling pressures. His electrocardiography was suggestive of sinus bradycardia with right bundle branch block.

The patient’s Society of Thoracic Surgeons Predicted Risk of Mortality (STS PROM) score was estimated at 5.9%, indicating intermediate-to-high surgical risk.^7^ Given prior VSARR and hemiarch reconstruction, redo surgical intervention was felt to carry increased technical complexity and operative risk. After a multidisciplinary Heart Team discussion, TAVR was pursued despite guideline limitations for native valve AR, as the patient was considered a poor candidate for redo root surgery. The patient provided informed consent after discussion of the possible treatment options.

**Figure 1:**
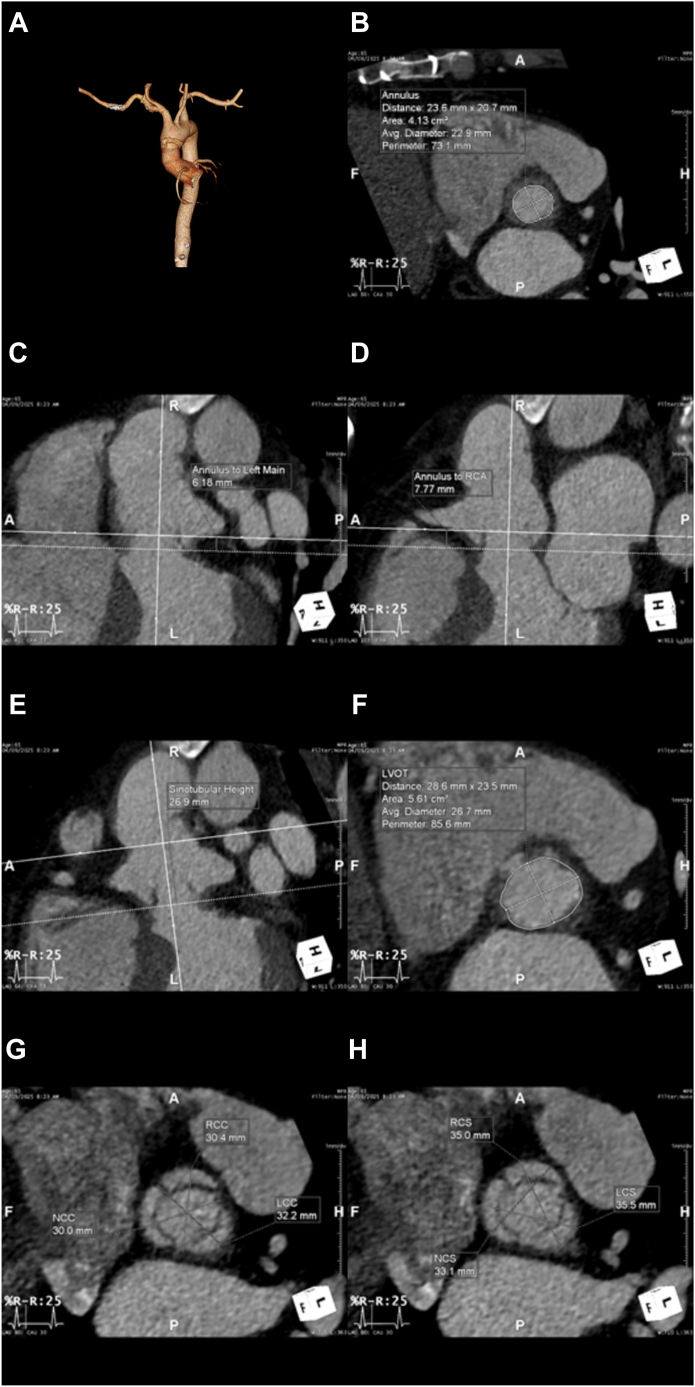
**Preprocedural computed tomography (CT) angiography.** (A) Multiplanar reconstruction from contrast-enhanced CT showing aortic root reconstructed with a Dacron graft from prior valve-sparing aortic root replacement. (B) The aortic annulus dimensions, with no significant annular calcification. (C-H) Coronary ostial heights, coronary sinus, and LVOT dimensions were adequate. LCC, left coronary cusp; LCS, left coronary sinus; LVOT, left ventricular outflow tract; NCC, non coronary cusp; NCS, noncoronary sinus; RCC, right coronary cusp; RCS, right coronary sinus.

Preprocedural computed tomography-angiography using a TAVR protocol demonstrated suitable anatomy and favorable peripheral vasculature for transfemoral access (Figure 1). Based on imaging, with approximately 25% perimetry oversizing, a 29-mm Evolut FX+ valve (Medtronic) was positioned and deployed under rapid ventricular pacing. The valve deployment resulted in multiple episodes of aortic valve migration requiring recapture and repositioning. The valve was eventually deployed once satisfactory positioning was confirmed in both the cusp overlap and left anterior oblique projections. Repeat angiography revealed ventricular migration of the Evolut valve with severe AR. As such, valve-in-valve TAVR using a 26-mm SAPIEN 3 Ultra valve (Edwards Lifesciences) was pursued. Wire position across the Evolut FX+ valve was maintained.

**Figure 2:**
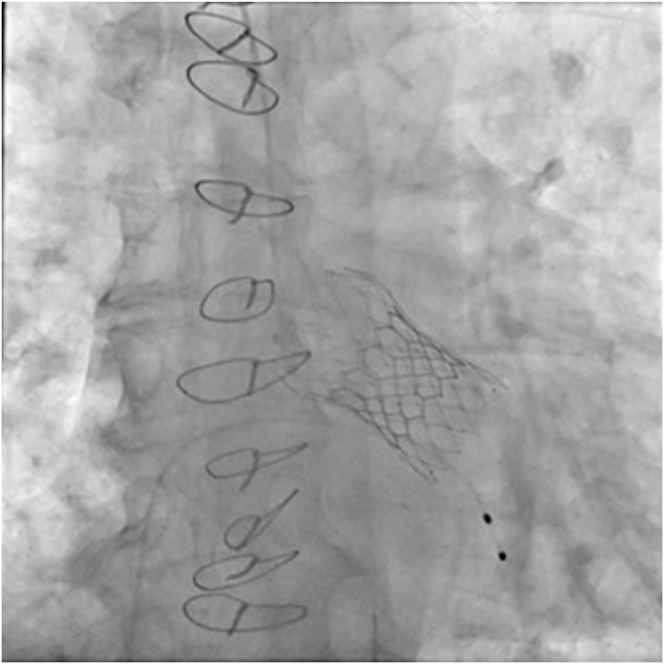
**Fluoroscopy image after deployment of the second valve-in-valve.**

After exchanging for a 14F expandable sheath, the SAPIEN valve was introduced, subsequently positioned across the aortic valve and successfully deployed (+1 cc volume) under rapid ventricular pacing (Figure 2, Supplemental Material).

Complete heart block requiring a pacemaker was noted after the second valve deployment. The patient tolerated the procedure well. A dual-chamber permanent pacemaker was implanted promptly after surgery.

The patient had an uneventful recovery. Postprocedural echocardiography showed a 26-mm Sapien 3 valve in proper position with no aortic valve prosthetic regurgitation, no periprosthetic or interprosthetic regurgitation with a mean gradient of 8 mm Hg and valve area 2.6 cm^2^, no pericardial effusion, and preserved LVEF. He was discharged home the next day on anticoagulation therapy and guideline-directed medical therapy for heart failure.

## Discussion

Valve-sparing aortic root replacement preserves native valve function and avoids long-term anticoagulation; however, it fundamentally alters aortic root geometry and compliance. The loss of native sinuses, tissue elasticity, and calcific anchoring creates a uniquely challenging environment for THV fixation.^8^ In this setting, valve stability relies predominantly on radial force, device oversizing, and friction between the THV frame and the noncompliant synthetic graft.^9^ These challenges are compounded in patients with minimal leaflet calcification. As a result, transcatheter valve migration remains a recognized and potentially catastrophic complication. Ventricular migration may result in acute severe regurgitation with rapid hemodynamic compromise, necessitating immediate intervention.^5^^,^^6^

In the present case, ventricular migration occurred shortly after deployment despite careful preprocedural planning. Prompt recognition allowed for successful valve-in-valve rescue using a balloon-expandable THV, which provided enhanced radial strength and controlled deployment within the migrated valve frame.

Recent efforts to improve patient selection and procedural planning for TAVR in pure AR, including anatomical classifications based on dual anchoring theory such as the AURORA framework, further highlight the importance of anchoring mechanics in noncalcified anatomy. This case underscores the procedural risks inherent to TAVR in a prosthetic aortic root and emphasizes the importance of preparedness for rapid valve-in-valve rescue when treating highly selected patients.^10^

## Conclusion

This case highlights the unique challenges and risks associated with TAVR in patients with prior VSARR. The absence of calcification and presence of a synthetic graft create a high-risk environment for THV malposition and migration. Second valve implantation proved to be an effective bailout strategy, restoring valve performance and stabilizing the patient. Careful preprocedural planning, consideration of altered surgical and anatomical landmarks, and preparedness for bailout techniques are essential for optimizing outcomes in this complex and evolving subset of patients.

## Declaration of competing interest

The authors have no potential conflicts of interest with respect to the research, authorship, and/or publication of this article.

## Supplementary material

Supplementary Data Presentation: Cine runs showing device migration and deployment of the second valve-in-valve in a patient with prior valve-sparing aortic root replacement.

## Footnotes

To access the supplementary material accompanying this article, visit the online version of the *Journal of the Society for Cardiovascular Angiography & Interventions* at 10.1016/j.jscai.2026.105344.
