# Supplementary material for: Ventricular Migration of Transcatheter Valve During Transcatheter Aortic Valve Replacement After Valve-Sparing Root Replacement: A Case Report
Source: J Soc Cardiovasc Angiogr Interv. 2026 Apr 30;5(6):105344. doi: 10.1016/j.jscai.2026.105344 (PMC13404056; doi:10.1016/j.jscai.2026.105344)
Supplement: Supplementary Data — Presentation: Cine runs showing device migration and deployment of the second valve-in-valve in a patient with prior valve-sparing aortic root replacement. [file mmc1.pptx]

## Slide 1
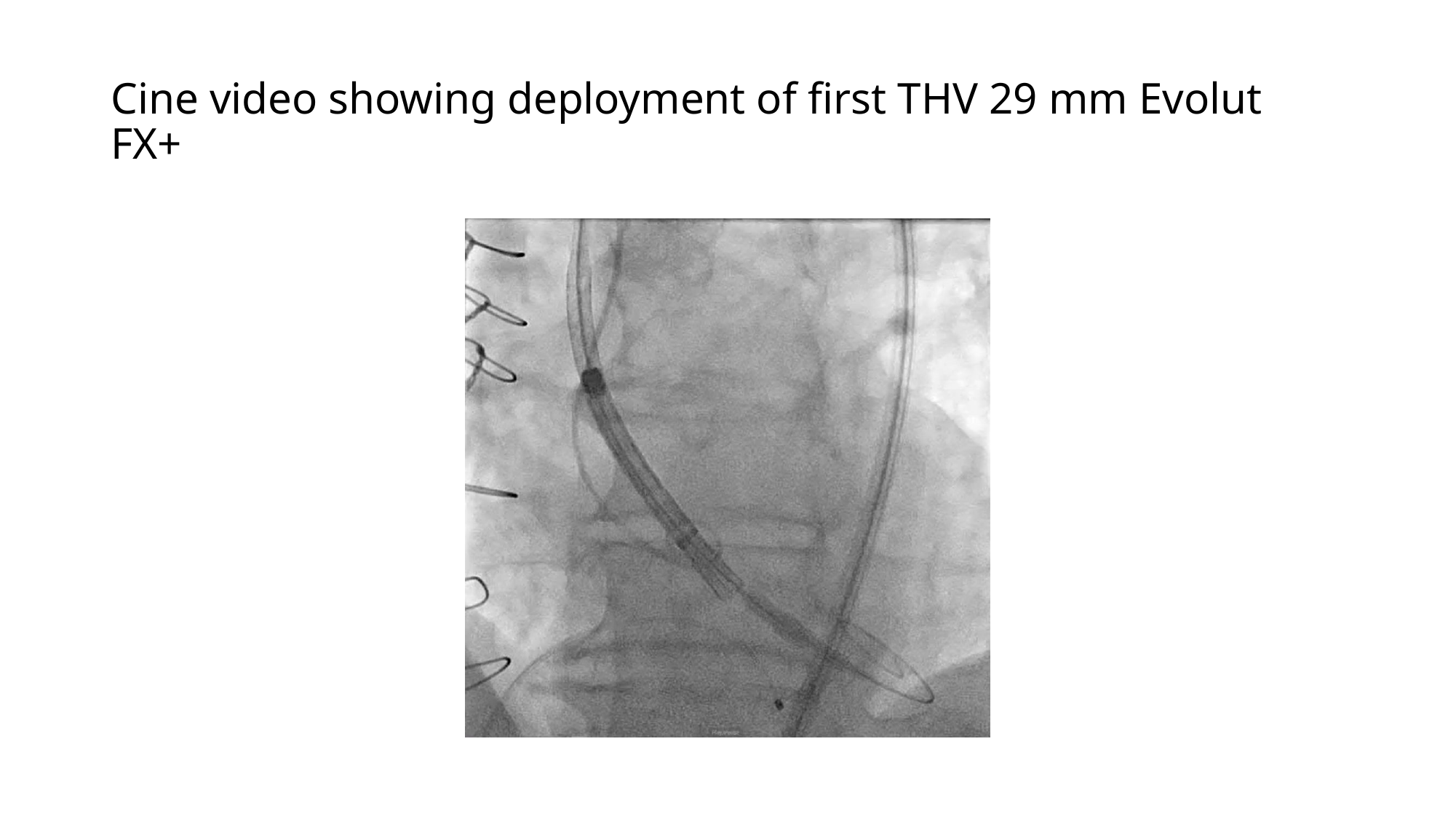

# Cine video showing deployment of first THV 29 mm Evolut FX+

## Slide 2
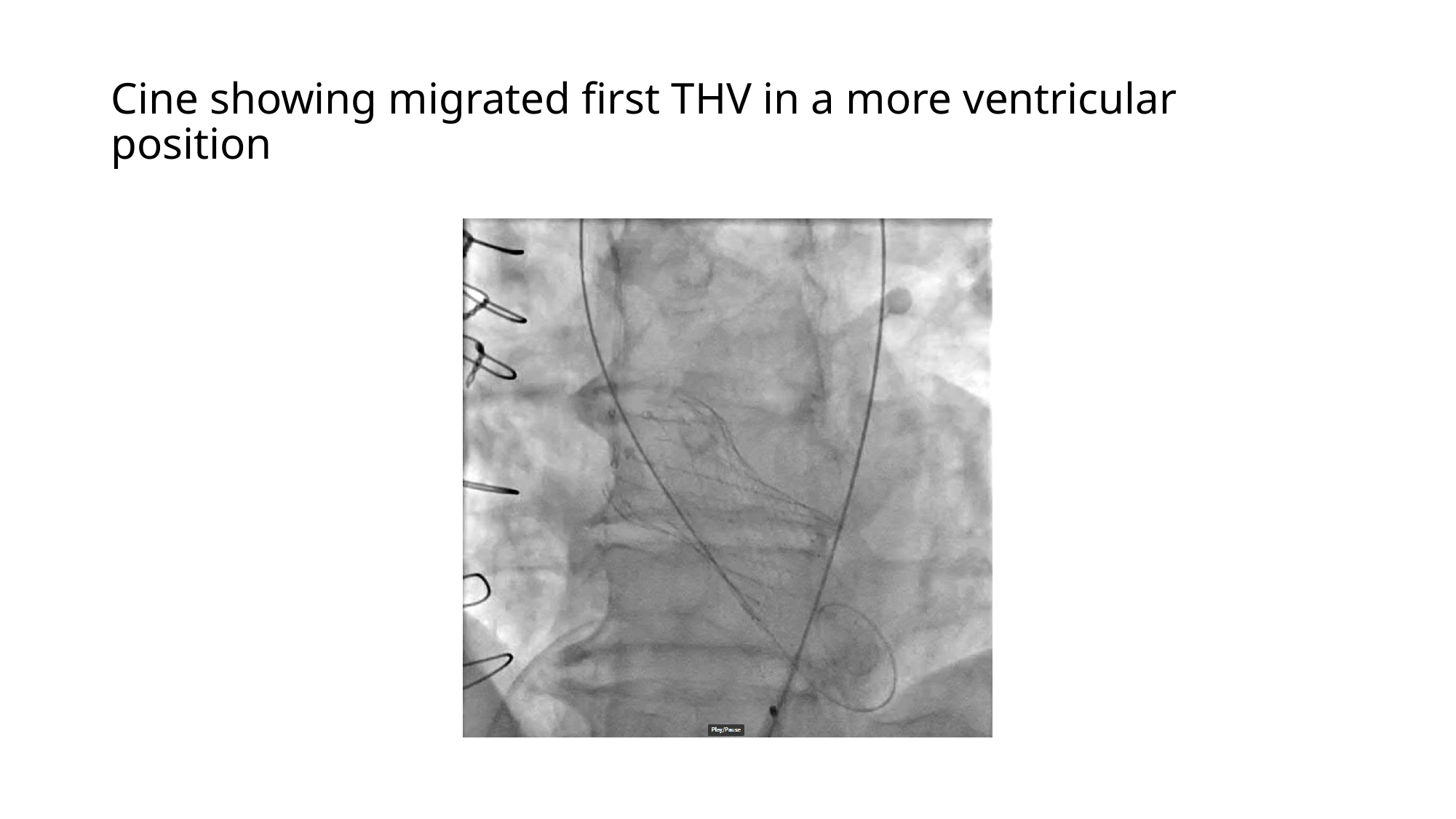

# Cine showing migrated first THV in a more ventricular position

## Slide 3
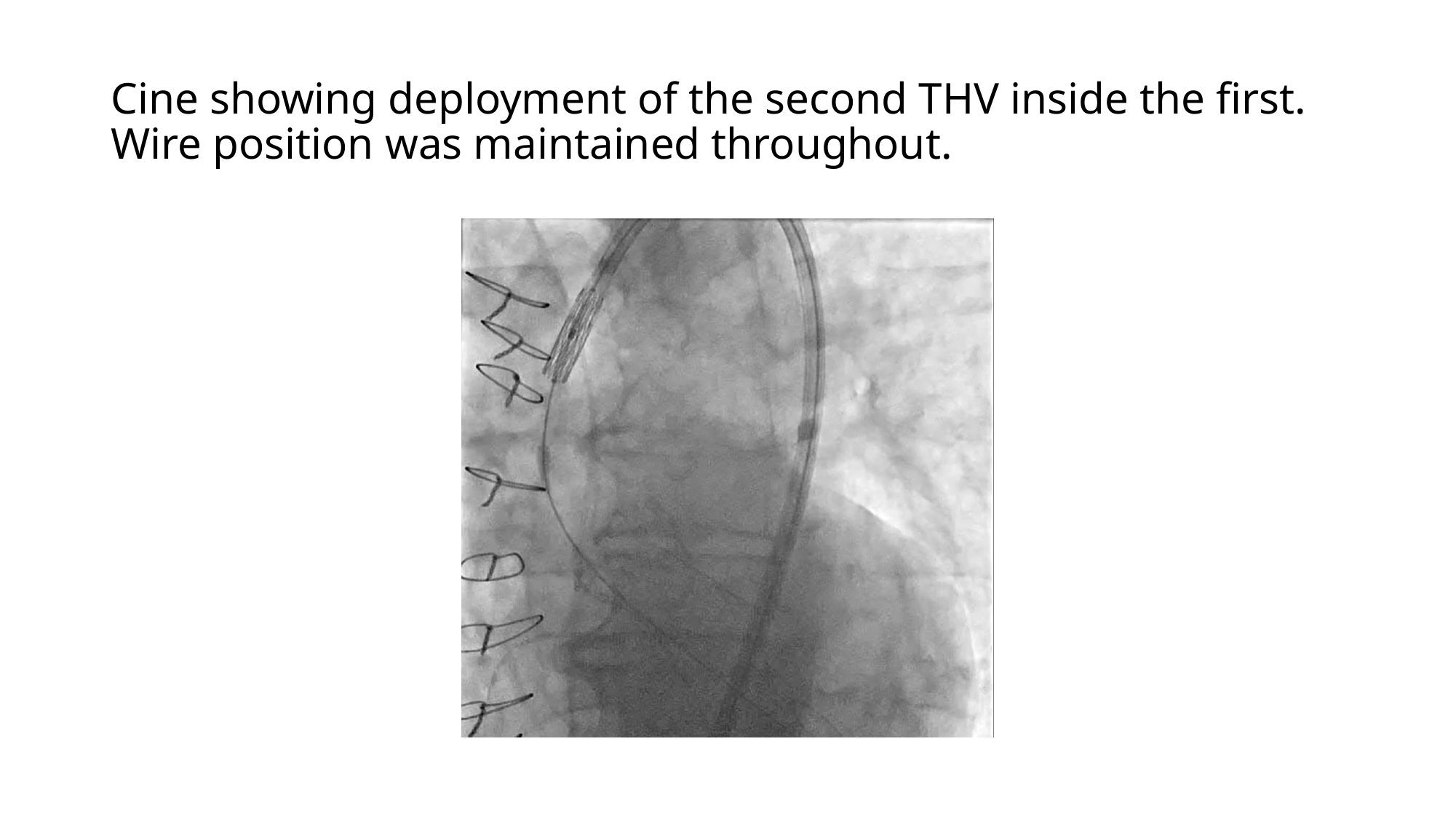

# Cine showing deployment of the second THV inside the first. Wire position was maintained throughout.

## Slide 4
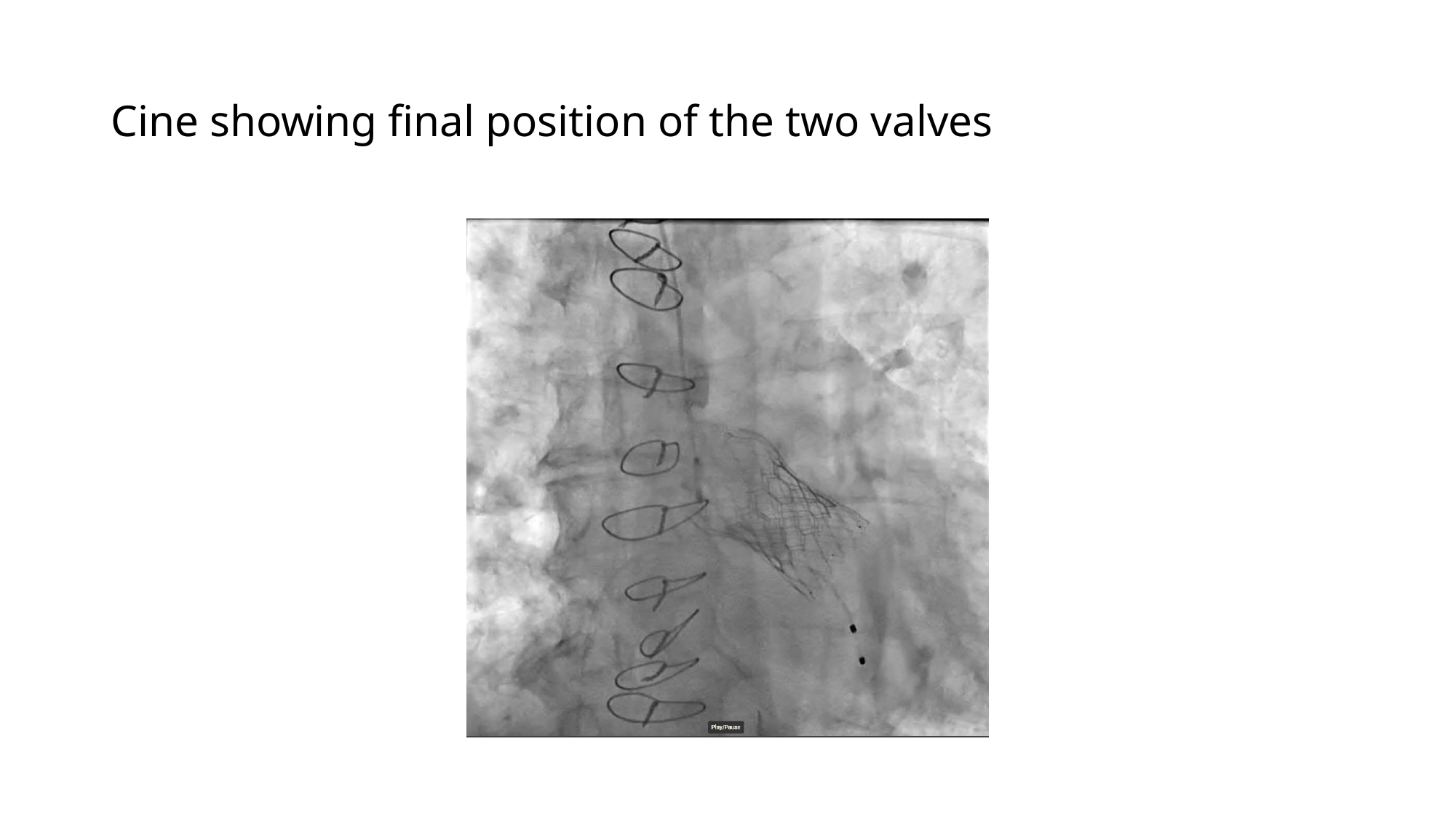

# Cine showing final position of the two valves
